# *BiomeHorizon*: Visualizing Microbiome Time Series Data in R

**DOI:** 10.1128/msystems.01380-21

**Published:** 2022-05-05

**Authors:** Isaac Fink, Richard J. Abdill, Ran Blekhman, Laura Grieneisen

**Affiliations:** a Department of Computer Science, University of Chicago, Chicago, Illinois, USA; b Department of Genetics, Cell Biology, and Development, University of Minnesota, Minneapolis, Minnesota, USA; c Department of Ecology, Evolution and Behavior, University of Minnesota, St. Paul, Minnesota, USA; Dalhousie University

**Keywords:** R package, microbiome, time series

## Abstract

Despite playing a key role in the health of their hosts, host-associated microbial communities demonstrate considerable variation over time. These communities comprise thousands of temporally dynamic taxa, which makes visualizing microbial time series data challenging. As such, a method to visualize both the proportional and absolute change in the relative abundance of multiple taxa across multiple subjects over time is needed. To address this gap, we developed *BiomeHorizon*, the first automated, open-source R package that visualizes longitudinal compositional microbiome data using horizon plots. *BiomeHorizon* is available at https://github.com/blekhmanlab/biomehorizon/ and a guide with step-by-step instructions for using the package is provided at https://blekhmanlab.github.io/biomehorizon/.

**IMPORTANCE** Host-associated microbiota (i.e., the number and types of bacteria in the body) can have profound impacts on an animal’s day-to-day functioning as well as their long-term health. Recent work has shown that these microbial communities change substantially over time, so it is important to be able to link changes in the abundance of certain microbes with host health outcomes. However, visualizing such changes is difficult because the microbiome comprises thousands of different microbes. To address this issue, we developed *BiomeHorizon*, an R package for visualizing longitudinal microbiome data using horizon plots. *BiomeHorizon* accepts a range of data formats and was developed with two common microbiome study designs in mind: human health studies, where the microbiome is sampled at set time points, and observational wildlife studies, where samples may be collected at irregular time intervals. *BiomeHorizon* thus provides a flexible, user-friendly approach to microbiome time series data visualization and analysis.

## INTRODUCTION

Despite playing a key role in the health of their hosts (1, 2), host-associated microbial communities demonstrate considerable variation both between hosts and within an individual host over time (3–5). To determine drivers of this temporal variation, and to link such variation to specific host health outcomes, recent work has focused on collecting time series microbiome samples from individual hosts. However, host-associated microbiome data are compositionally complex, with thousands of microbial taxa present at any given time point, and visualizing these longitudinal data is challenging. Traditional methods of longitudinal microbiome visualization use a stream or line graph with a different color for each microbe (5–8). While this is valuable for tracking a single microbe in a single host, it becomes visually difficult to distinguish broader trends among several microbes, or to compare microbial trends at the same time points across multiple hosts. This is especially true given that large proportional changes in microbes with low abundances (e.g., mean relative abundance <0.5%) are dwarfed by highly abundant microbes (e.g., mean abundance >25%). Further, line graphs do not facilitate comparing microbial trends at the same time points across different hosts. Hence, tools that enable automated visualization of proportional changes across microbes, hosts, and/or time are crucial for exploratory data analysis, as well as for detailed comparisons of select taxa, individuals, or time slots.

## RESULTS

### Overview.

Here, we present *BiomeHorizon*, a *ggplot2*-compatible R package that provides an automated way of visualizing the longitudinal dynamics of multiple microbes in parallel (9). *BiomeHorizon* was developed with two of the most common microbiome study designs in mind: human health experiments, such as dietary or medical intervention studies, where the microbiome is sampled from all subjects at set time points to compare microbiome health outcomes over time; and observational wildlife studies, where samples may be collected at irregular time intervals (e.g., opportunistically when defecation is observed) and/or with large time gaps.

**FIG 1:**
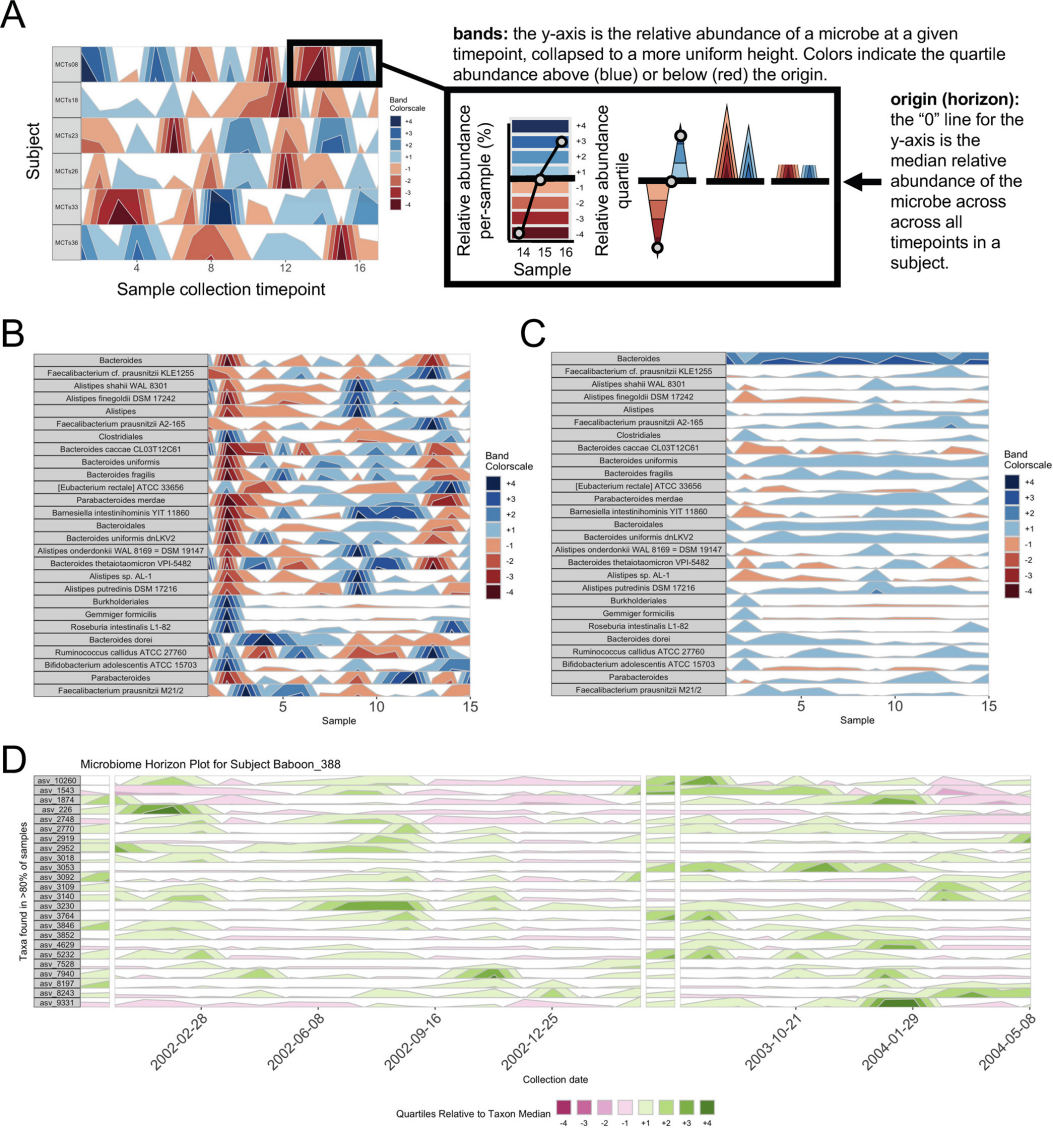
A *BiomeHorizon* horizon plot showing custom configurations. (A) Annotated horizon plot for a single microbe (single_var_otu = “Taxon 1”), for 17 samples across 6 subjects in the diet study example data set, illustrating how a horizon plot is constructed. First, values are plotted as a relative abundance versus time area graph for each OTU time series. Values are then centered to a “zero,” in this case the median relative abundance. This centered value is referred to as the “horizon” or “origin.” Next, the plotting area is divided into quartile “bands” above and below the origin, with darker blue bands indicating values incrementally above the origin and darker red bands below the origin; negative bands are mirrored upward. Finally, bands are overlaid to compress vertical space. (B) Microbes manually chosen as those with a per-sample average relative abundance of at least 0.75% (thresh_abundance = 0.75) across 15 samples in one subject (subj = “MCTs01”) in the diet study example data set. Microbes are labeled by their most fine-grained level of taxonomic identification (facetLabelsByTaxonomy = TRUE). (C) The same data as B, but with the origin manually set to 1% relative abundance (origin = 1) and band thickness set so each band represents 10% relative abundance (band.thickness = 10), which serves to visually emphasize changes in highly abundant microbes (e.g., *Bacteroides*). (D) For data collected at irregular time intervals or with collection gaps (shown in the wild baboon study example data set), *BiomeHorizon* can interpolate between points to regularize intervals (25 days shown here; regularInterval = 25) with breaks when there are gaps greater than a specified interval (75 days shown here; maxGap = 75). Custom aesthetics can be used to adjust labels, colors, etc.

*BiomeHorizon* generates horizon plots, charts in which the *x* axis is a time series and the *y* axis starts from the “horizon” (or “origin,” often the median value of a variable across all time points) and features areas whose *y* axis distribution represents the distance of the variable from the origin at a given time point (Fig. 1A) (10). Colored bands are used to represent *n*-tiles from the origin, with two different color families representing positive or negative values. The compactness of horizon plots facilitates pattern-recognition and comparison between numerous time series, revealing unique insights into longitudinal data. Specifically, they allow visual identification of sustained versus temporary change in microbe(s), which is valuable for modeling stability and disturbance (6). For example, it is easy to detect “comovement” or “periodicity,” while also comparing amplitude. Comovement can be valuable for identifying microbes with related functions (e.g., similar proportional increases at the same time points may indicate taxa play similar roles for the host), while periodicity might reveal links between environmental or experimental factors and microbial dynamics (6).

*BiomeHorizon* is the first horizon plot tool specific to microbiome data. As such, it provides three innovations specific to microbiome data compared to prior horizon plot applications, which also allow substantial room for customization and adaptation (11, 12). First, it takes data in a common OTU table format, as either read counts or per-sample relative abundance, with functions to filter taxa based on prevalence and abundance. We note that “OTU” table here refers to the finest level of microbial taxonomic delineation available in the input data. This table can be generated from amplicon sequencing output, or alternatively, from shotgun metagenomic output. For example, for 16S rRNA gene amplicon input data, the input table can be comprised of actual OTUs (operational taxonomic units created by clustering amplicons at some percentage identity threshold) or of ASVs (amplicon sequence variants). For metagenomic shotgun data, the input table could mean a sequence with taxonomic identity assigned via BURST (5), or to an actual OTU obtained by extracting the marker genes (e.g., using a program like PhyloFlash [13]). For simplicity, we will continue to use the term “OTU” throughout this paper, as that is the term used in the *BiomeHorizon* package itself. A taxonomy table and metadata table may be supplemented such that microbes can be annotated by taxonomic level and selected from subject(s) of interest, respectively. These data sets are accepted in a variety of formats (see online tutorial for full examples, including acceptable variable names), making the package applicable to a wide range of experimental and observational conditions.

Second, *BiomeHorizon* can simultaneously compare abundant and rare taxa by showing proportional changes. Specifically, the tool can switch between a fixed origin or *y* axis scale for each microbe, and variable origin or *y* axis scale, allowing for comparisons between multiple microbes and between multiple hosts. The user can also customize the number of bands. Including more horizon bands will more precisely distinguish values and emphasize those at the highest ranges of magnitude, while using fewer bands will de-emphasize values at the extreme ends of the data.

Third, *BiomeHorizon* can accurately reflect taxa dynamics across irregular time intervals, making it suitable for visualizing data sets with lengthy gaps. These customizations make *BiomeHorizon* versatile in highlighting a wide range of aspects of longitudinal data, and facilitate the user’s ability to pick which microbes may be of interest for additional analyses.

### Usage scenario.

To demonstrate the versatility of *BiomeHorizon*, we apply it to two different publicly available microbiome data sets: a 17-day human diet experimental study with metagenomic sequencing of the gut microbiome (5), and a multiyear collection of wild baboon 16S rRNA gut microbiome samples (14). By using single_var_otu, *BiomeHorizon* can compare the temporal dynamics of a single microbe across multiple subjects with samples collected on the same days (Fig. 1A).


## Subset the data set to the subjects who were sampled on all 17 days, and arrange by date
metadata_17 <− metadatasample_diet %>%
filter (subject %in% c("MCTs08", "MCTs18", "MCTs23", "MCTs26", "MCTs33",
"MCTs36")) %>%
arrange(subject, collection_date)
otu_17 <− otusample_diet %>%
select(taxon_id, as.character((metadatasample_diet %>% filter(subject %in% c
("MCTs08", "MCTs18", "MCTs23", "MCTs26", "MCTs33", "MCTs36")))$sample))
## Single variable analysis with "Taxon 1"
paramList <− prepanel(otudata = otu_17, metadata = metadata_17,
singleVarOTU = "taxon 1")
horizonplot(paramList)


Alternatively, by adjusting thresh_prevalence, thresh_abundance, or otulist, and specifying subj, microbes can be filtered by prevalence and abundance, or by name, to compare many microbes in the same subject (Fig. 1B).


paramList <− prepanel(otudata = otusample_diet, metadata = metadatasample_diet,
taxonomydata = taxonomysample_diet, $taxonomy, subj = "MCTs01",
facetLabelsByTaxonomy = TRUE, thresh_abundance = 0.75)


Further, by adjusting origin or band.thickness, the dynamics of highly abundant or rare microbes can be emphasized (Fig. 1C).


paramList <− prepanel(otudata = otusample_diet, metadata = metadatasample_diet,
taxonomydata = taxonomysample_diet$taxonomy, subj = "MCTs01",
facetLabelsByTaxonomy = TRUE, origin = 1, band.thickness = 10,
thresh_abundance = 0.75)



The origin is the base of the first positive band for an OTU, where the *y* axis value = 0. The y-scale height of each band is the band thickness. By default, the origin for each OTU is calculated as the median value of that OTU across all samples, and band thickness represents 4 quartiles above (blue bands) and 4 quartiles below (red bands) the origin relative to the absolute extreme value for that OTU. By scaling within each OTU, the dynamics of multiple OTUs that may vary in median abundance by orders of magnitude can be visualized on the same graph. At smaller values of band.thickness, an increasing number of values are above the new maximum or below the new minimum, resulting in more extreme bands (at +4 or −4). This accentuates changes in microbes with low abundances but compresses change in microbes with larger abundances.

Finally, for data at irregular time intervals, such as those collected in the wild baboon example data set, regularInterval specifies the interval at which missing data can be interpolated. The new, interpolated point is calculated based on a linear interpolation between the previous and subsequent observed time points. maxGap specifies the maximum amount of time between sample collection before a gap in the *x* axis rather than an interpolated point should be used (Fig. 1D).


paramList <− prepanel(otudata = otusample_baboon,
metadata = metadatasample_baboon, subj = “Baboon_388”,
regularInterval = 25, maxGap = 75)


## DISCUSSION

*BiomeHorizon* is a powerful tool for visualization of microbiome dynamics over time, as well as a useful initial data exploration tool. It is highly customizable and versatile, as it is designed to accommodate both metagenomics and 16S microbiome data, and can be easily integrated into *ggplot2*, allowing for esthetic customization. Although it is designed for microbiome data, *BiomeHorizon* can also be applied to other types of longitudinal data sets that are represented as the relative abundance of many features. We also note that changes in the relative abundance of a feature can occur despite a stable absolute abundance (i.e., if one feature increases in absolute abundance, other features will show a change in relative abundance), so follow-up statistical analyses to initial *BiomeHorizon* explorations are recommended. *BiomeHorizon* is an open-source project available on GitHub, with a tutorial to supplement the documentation.

## MATERIALS AND METHODS

### Implementation.

BiomeHorizon is available at https://github.com/blekhmanlab/biomehorizon/ and released under the MIT license. The submitted software version is archived on Zenodo at https://doi.org/10.5281/zenodo.5469141. A guide with step-by-step instructions for using the package is provided at https://blekhmanlab.github.io/biomehorizon/. The guide also provides code to reproduce all plots in this manuscript.

### Data availability.

The full version of the data sets used in this article and incorporated into the *BiomeHorizon* package are available on Zenodo (baboon data set at https://doi.org/10.5281/zenodo.4662081) and Github (human diet data set at https://github.com/knights-lab/dietstudy_analyses/tree/master/data/maps).
